# Influence of Bioactive Glass Incorporation in Resin Adhesives of Orthodontic Brackets on Adhesion Properties and Calcium Release

**DOI:** 10.3390/polym17172282

**Published:** 2025-08-23

**Authors:** Ana Paula Valente Pinho Mafetano, Fernanda Alves Feitosa, Gabriela da Silva Chagas, Nathália Moreira Gomes, Marcella Batista Rocha, Mariane Cintra Mailart, Karen Cristina Kazue Yui, Cesar Rogério Pucci

**Affiliations:** 1 Department of Restorative Dentistry, Institute of Science and Technology, São Paulo State University (UNESP), São José dos Campos 12245-000, SP, Brazil; ana-paula.mafetano@unesp.br (A.P.V.P.M.); gabriela.chagas@unesp.br (G.d.S.C.); nathalia.m.gomes@unesp.br (N.M.G.); marcella.b.rocha@unesp.br (M.B.R.); mariane.cintra-mailart@unesp.br (M.C.M.); 2 Department of Pediatric and Social Dentistry, Institute of Science and Technology, São Paulo State University (UNESP), São José dos Campos 12245-000, SP, Brazil; karen.yui@unesp.br

**Keywords:** bioactive glass particles, orthodontics, shear bond test, white spot lesion

## Abstract

This study evaluated a light-cure orthodontic adhesive with the incorporation of bioactive glass particles and its effects on shear bond strength (SBS), adhesive remnant index (ARI), degree of conversion (DC), calcium release, and particle size distribution. Bioactive glass was added to the Transbond XT Adhesive (3M ESPE), resulting in five groups: TXT (0% wt of bioactive glass-incorporated—negative control); TXT20 (20% wt of bioactive glass-incorporated); TXT30 (30% wt of bioactive glass-incorporated), TXT50 (50% wt of bioactive glass-incorporated), and FLB (positive control—FL BOND II adhesive system with S-PRG particles, SHOFU Inc.). Data were analyzed with one-way ANOVA followed by Tukey’s test (α = 0.05). Quantitative SEM analysis confirmed submicron particle agglomerates (median equivalent circular diameter 0.020–0.108 µm). The TXT20 exhibited the highest values of degree of conversion (*p* < 0.05) (73.02 ± 3.33A). For SBS (in MPa): Control Group TXT—19.50 ± 1.40A, Group TXT20 18.22 ± 1.04AB, Group FLB 17.62 ± 1.45B, Group TXT30 14.48 ± 1.46C and Group TXT50 14.13 ± 1.02C (*p* < 0.05). For calcium release the group TXT50 2.23 ± 0.11D showed higher values (*p* < 0.05). The incorporation of distinct bioactive glass particle concentrations influenced the shear bond strength, degree of conversion, and calcium release. While the 50 wt% bioactive glass group exhibited the highest calcium release, both 20 wt% of bioactive glass group and the positive control group exhibited the highest degree of conversion without compromising the bonding strength.

## 1. Introduction

Maintaining proper oral hygiene is one of the greatest challenges faced by patients during orthodontic treatment. The use of bonding materials and brackets fixed to the tooth surface facilitates the retention of biofilm, leading to greater biofilm retention [1,2,3]. Combined with high carbohydrate diets and the presence of cariogenic bacteria, this can lead to white spot lesions (WSLs) [4,5,6,7]. In the early stages, the lesions are chalky, and opaque areas around the brackets are observed. These are referred to as white spot lesions (WSL).

The WSL has the potential to become irreversible, as they affect areas that are difficult to clean, which may also impact the patient’s overall esthetic appearance [8,9]. These lesions are observed in 15.5% to 40% of patients before orthodontic treatment and in 30% to 70% during treatment [10]. A 45.8% incidence of new carious lesions has been observed in patients undergoing orthodontic treatment, with a prevalence rate of 68.4% [10,11]. In addition to the mechanical retention of biofilm and the difficulty in maintaining oral hygiene, changes in the oral bacterial flora during orthodontic treatment might lead to an increase in cariogenic bacteria levels. Patients with elevated levels of Streptococcus mutans and *Lactobacillus* before treatment are at a high risk of developing carious lesions throughout orthodontic therapy [12,13]. These bacteria can reduce the pH of biofilm through the metabolism of fermentable carbohydrates, leading to acid release [14,15].

Despite advances in preventive techniques, WSL prevention remains a significant clinical challenge. Standard recommendations include maintaining adequate oral hygiene, fluoride therapy, and diet control to reduce the risk of these lesions [15,16,17]. However, the effectiveness of hygiene recommendations relies on patient adherence to dental treatment, representing a challenge for dentists in assessing the protocol’s effectiveness outside the dental office. In this context, alternative approaches have been investigated, and the incorporation of bioactive glass particles into resin composites and adhesive systems has shown promising results in arresting initial caries lesions [18].

Bioactive glass particles are biomaterials composed of amorphous silicate, which are commonly used in bone regeneration, dentistry, and orthopedics [19]. The composition of bioactive glass particles varies, and the resulting antimicrobial effects, fluoride release, and apatite formation are of great importance in dentistry, particularly in the prevention of WSL [18,19,20]. Their ability to release ions from glass in solutions results in an increase in pH and the precipitation of calcium ions facilitates the formation of a mineral matrix that is analogous to natural hydroxyapatite and contributes to the increase in intraoral pH, helping to protect the tooth surface from wear by acids formed by cariogenic bacteria that metabolize fermentable carbohydrates, since the critical pH for enamel dissolution is 5.5 [20,21,22,23].

Although bioactive glass has been increasingly incorporated into resin-based mate-rials, the synergistic interactions between the resin matrix and bioactive glass particles remain poorly understood. Most studies have focused on ion release and antibacterial effects, whereas the influence of filler–matrix interactions on polymerization kinetics, chemical bonding, stress development, and long-term stability has not been fully elucidated [24,25]. Furthermore, the combined effect of resin monomer composition, particle surface chemistry, and filler concentration on these properties is still poorly understood, limiting the optimization of bioactive adhesive systems [25].

It is still unclear whether incorporating bioglass into orthodontic adhesives can prevent WSL formation around brackets. Accordingly, the objective of this study was to evaluate a light-cure orthodontic adhesive with the incorporation of bioactive glass particles and its effects on shear bond strength (SBS), adhesive remnant index (ARI), degree of conversion (DC) and calcium release. The null hypotheses tested were that the different concentrations of bioactive glass incorporation into the light-cure orthodontic adhesive would not be different regarding H01—shear bond strength; H02—degree of conversion of the adhesive; and H03—calcium release.

## 2. Materials and Methods

### 2.1. Adhesive Preparation

The manipulation of the commercial resin adhesive with added bioactive glass particles was performed using bioactive glass powder (Vitryxx^®^ Bioactive Glass Powder, SCHOTT AG, Landshut, Bayern, Germany). Both resin adhesive and the powder of bioactive glass particles were weighed, according to the concentrations, for each experimental group. Afterward, they were properly mixed (5 min at a speed of ~250 rpm) in a Vortex-mixer (Phoenix, Luferco, Araraquara, SP, Brazil) in a plastic container. To perform the treatments, the specimens were allocated to five groups:

- TXT (0% wt of bioactive glass-incorporated—negative control);
- TXT20 (20% wt of bioactive glass-incorporated);
- TXT30 (30% wt of bioactive glass-incorporated);
- TXT50 (50% wt of bioactive glass-incorporated);
- FLB (positive control—FL BOND II adhesive system with S-PRG biomaterial, SHOFU Inc. Kyoto, Japan)

Previous investigations have consistently demonstrated that the incorporation of up to 20 wt% bioactive glass into adhesive systems preserves or even enhances monomer conversion and bond strength, without adversely affecting the mechanical properties of the material [26]. Furthermore, the inclusion of 30 wt% bioactive glass was selected based on prior evidence indicating that formulations containing up to 30–40 wt% maintained a stable degree of conversion (DC) while providing additional benefits such as remineralizing potential and antibacterial activity [24]. Based on this evidence, the concentrations of 20 wt%, 30 wt%, and 50 wt% were chosen for the present study: the first two fall within previously validated and widely accepted ranges, while 50 wt% was incorporated as an exploratory condition. Table 1 shows the composition of tested materials.

### 2.2. Specimen Preparation

Fifty recently extracted bovine incisors with sound enamel and no visible enamel alterations were selected. The teeth were stored in 0.1% timol solution at 4 °C until the use [27,28]. The teeth had their crowns and roots standardized and were then embedded in ½-inch Polyvinyl Chloride (PVC) rings with the aid of a self-curing acrylic resin (Jet Classic, São Paulo, SP, Brazil). The buccal surface was aligned perpendicularly to the base of the PVC ring. The randomization of samples was performed.

### 2.3. Bonding Brackets to the Enamel Surface

The buccal surface of the teeth underwent prophylaxis using prophylactic brushes and pumice slurry for 30 s. The teeth were then etched with 37% phosphoric acid for 30 s, abundantly rinsed with deionized water for 30 s and gently air-dried using a syringe. One drop of the adhesive system for each tooth of each specific experimental group was actively applied using a brush, air-dried using a syringe to evaporate the solvent and subsequently light-cured for 20 s (Radii-Cal light-curing device, SDI, Bayswater, VIC, Australia), following the manufacturer’s instructions [29]. Then, Transbond XT light-cure orthodontic adhesive was used (0% wt, 20% wt, 30% wt and 50% wt of bioactive glass-incorporated) on the base of the self-ligating brackets (self-ligating steel bracket—SLI MORELLI, Sorocaba, SP, BR—Ref. 1014900) to bond them to the center of the clinical crown on the buccal surface of the bovine incisors.

### 2.4. Shear Bond Strength

The previously prepared fifty bovine incisors were randomly allocated into five groups (*n* = 10). The shear bond strength test was then conducted using an EMIC DL2000 universal testing machine (EMIC, São José dos Pinhais, PR, Brazil). A chisel was positioned on the upper part of the bracket and an occlusogingival load of 100 kgf at a speed of 1.0 mm/min was applied to the bracket, producing a shear load at the bracket-tooth interface until failure occurred. Shear strength is equal to the force required to detach the bracket from the enamel surface. After the test, the parts were analyzed under an optical stereomicroscope with 10× magnification (ZEISS, stereo Discovery V20, Gottingen, Germany) and classified according to the type of failure.

### 2.5. Analysis of the Index of Adhesive Residue Adhered to the Tooth After Bracket Detachment

The failure surfaces were classified according to the Adhesive Remnant Index (ARI) proposed by Årtün and Berglund in 1984 [30], which quantifies the amount of resin remaining on the enamel to identify the site of fracture during the bond strength test. After bracket debonding, ARI scores were determined using a stereoscopic magnifying glass (Carl Zeiss, São Paulo, SP, Brazil) at 10× magnification by a single blind operator. The evaluation of residual adhesive on the enamel was performed in accordance with the scoring system described by Årtün and Berglund:

- Score 0—no remaining amount of composite adhered to the tooth;
- Score 1—less than half the amount of composite adhered to the tooth;
- Score 2—more than half the amount of composite adhered to the tooth;
- Score 3—all the composite adhered to the tooth amount of composite adhered to the tooth.

### 2.6. Degree of Conversion

The degree of conversion (DC) was measured by Fourier Transform Infrared Spectroscopy (FTIR) by the software Spectrum TimeBase 10 (Perkin Elmer, Beaconsfield, UK). The samples of the light-cured orthodontic adhesives with and without the incorporation of bioactive glass particles were analyzed by absorbance with the attenuated total reflectance (ATR) device with a zinc selenide (ZnSe) diamond crystal plate (PIKE Technologies, Madison, WI, USA). Initially, a small amount, 3 µL, of the TXT (0% wt, 20% wt, 30% wt and 50% wt) and FLB adhesives was placed on the ATR crystal using a pipette (LambdaTM Plus, Corning Co., Corning, NY, USA), the amount necessary for the adhesive to wet the entire surface of the crystal in a yellow light environment. Absorbance data were immediately collected using the spectral range from 4000 to 650 cm^−1^ was used to obtain the spectra of the uncured adhesive and the corresponding polymerized adhesive, with the resolution of 4 cm^−1^ and 32 captures. A change in the band height ratios of the aliphatic carbon–carbon double bond (peak at 1638 cm^−1^) and aromatic C=C (phenyl peak at 1608 cm^−1^) was observed in the cured and uncured states. The adhesive sample was photoactivated with the Radii-Cal photopolymerizer (SDI, Bayswater, VIC, Australia) at a distance of approximately 5 mm. To calculate the degree of conversion (DC), the formula based on the decrease in the intensity of the band ratios before and after light curing was used (Equation (1)).

(1) $$DC\;\left(\%\right)=\left(1-\left(\frac{Rcured}{Runcured}\right)\right)\times 100$$

All experiments were carried out in triplicate, and the results were averaged.

### 2.7. Calcium Release

A buffered sodium chloride solution at pH 4.0 with 50 mmol/L acetic acid was used. The pH 4.0 represents an acidic environment, useful for testing the maximum calcium-releasing capacity of the material, as can occur in caries lesions or under bacterial plaque [31]. Circular specimens of adhesives (diameter = 6 mm and thickness = 1.5 mm) were prepared using custom molds. The adhesive disks were light cured for 20 s from each side using the Radii-Cal (SDI). The samples were divided into five groups (n = 3) and immersed in the buffered sodium chloride solution with acetic acid where they were stored at 37 °C. Calcium release was measured using the arsenazo III method (Fluitest 1, Ca-A-II, analyticon, Lichtenfels, Bavaria, Germany) [32,33]. Arsenazo III reacts with calcium in the acid solution to form a blue complex, and the intensity of the reaction is proportional to the calcium concentration. The absorbance of calcium release was analyzed by ICP-OES (Inductively Coupled Plasma Optical Emission Spectrometry—ICPE-9820, Shimadzu, Shimadzu do Brasil, Barueri, SP, Brazil) at the optimum wavelength of 650 nm in the radial view. Readings of the solutions were performed in duplicate for each experimental group and measured at 7th day, with results expressed in mg/L.

### 2.8. Scanning Electron Microscopy (SEM)

The morphology of the bioactive glass particles was analyzed by scanning electron microscopy (SEM) at magnifications of 1000× and 1500×. The samples were mounted on aluminum stubs using double-sided carbon adhesive tape, sputter-coated with a thin layer of gold/palladium (Desk II, Denton Vacuum, Moorestown, NJ, USA), and examined in a scanning electron microscope (JSM-5310, Jeol Ltd., Tokyo, Japan) operated at an accelerating voltage of 20 kV.

### 2.9. Quantitative Analysis of SEM

Calibrated SEM micrographs were analyzed using ImageJ Fiji 1.54p software (National Institute of Health, Bethesda, MD, USA). Images were converted to 8-bit, background-subtracted, thresholded (Otsu), and segmented using watershed. Border-touching objects were excluded. For each particle, area, perimeter, circularity, Feret diameter, and equivalent circular diameter (ECD = 2√(area/π)) were computed. Given the visible agglomeration, results are reported as projected agglomerate size distributions (mean ± SD, median [IQR], and D10/D50/D90 percentiles). Dispersion within fields was additionally assessed by particle area fraction and the coefficient of variation (CV) across fields.

### 2.10. Statistical Analyses

Sample size for shear bond strength was calculated using GPower 3.1 (Heinrich-Heine-University, Düsseldorf, Germany) based on a pilot study effect size (Cohen’s f = 1.25). For a one-way ANOVA with 5 groups, α = 0.05, and power = 0.80, the required sample size was 5.98 per group. To ensure robustness and account for potential losses, n = 10 per group (total N = 50) was used. For the calcium release assay, a priori sample size calculation using GPower 3.1 was performed for a one-way ANOVA with 5 groups, α = 0.05, and power = 0.80. Assuming a large effect size (Cohen’s f = 0.40), the total required sample size was 15 (n = 3 per group). Data were tested for normality (Shapiro–Wilk) and homoscedasticity (Levene). Since data were normally distributed with homogeneous variances, degree of conversion, µTBS, and calcium release were analyzed by one-way ANOVA followed by Tukey’s post hoc test. Statistical analyses were performed using Statistica for Windows, version 8.0. Adhesive remnant index data were tabulated by experimental group and analyzed using Fisher’s exact test to assess associations between groups and score distributions.

## 3. Results

### 3.1. Shear Bond Strength (MPa), Degree of Conversion (DC), Calcium Release and Scanning Electron Microscopy (SEM)

Table 2 shows the mean values of shear bond strength (*p* = 0.00), DC (*p* = 0.00), and calcium release obtained for each group (*p* = 0.00).

**Figure 1:**
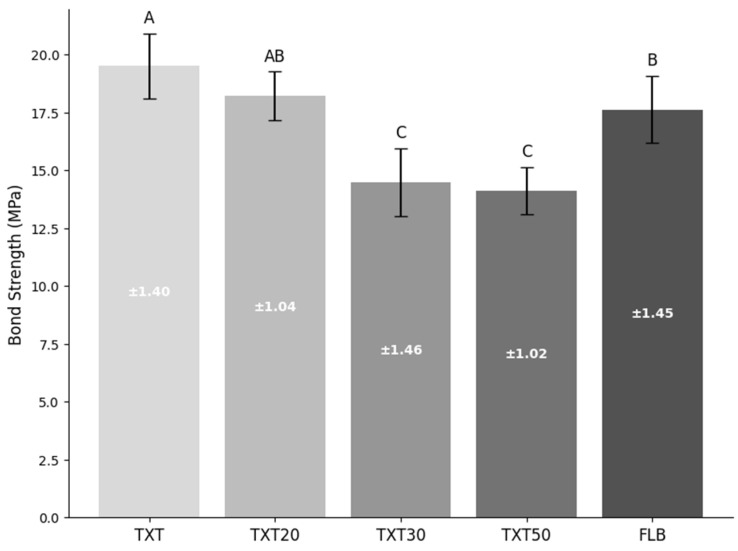
Mean and standard deviation of bond strength expressed in MegaPascal (Different upper-case letters show significant differences between the groups.).

The shear bond strength values were expressed in MPa. As observed in DC results, both TXT20 and FLB groups presented similar values and significantly higher than TXT30 and TXT50 groups. The highest values of shear bond strength were observed in the TXT group (Figure 1).

**Figure 2:**
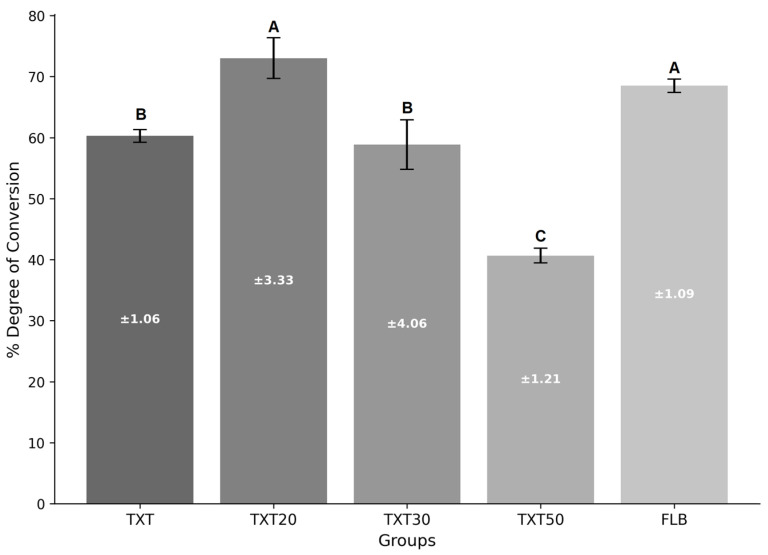
Mean and standard deviation of the percentage of Degree of Conversion of different adhesives (Different upper-case letters show significant differences between the groups.).

For DC (Figure 2), both TXT20 and FLB groups presented significantly higher values compared to the TXT and TXT30 groups. On the other hand, the lowest DC value was found for the TXT50 group.

Regarding calcium release, the TXT50 group exhibited the highest concentration of calcium release, followed by the TXT30 group. Both TXT20 and FLB groups showed similar calcium concentration values. The TXT group exhibited calcium concentration very close to zero.

### 3.2. Adhesive Remnant Index

After debonding, the adhesive remnant index (ARI) was evaluated according to Årtün and Bergland [30] criteria. Fisher’s exact test was conducted (*p* = 0.0245). Table 3 shows the adhesive remnant index (ARI) and the frequency of appearance of the scores analyzed in each group.

### 3.3. Scanning Electron Microscopy

**Figure 3:**
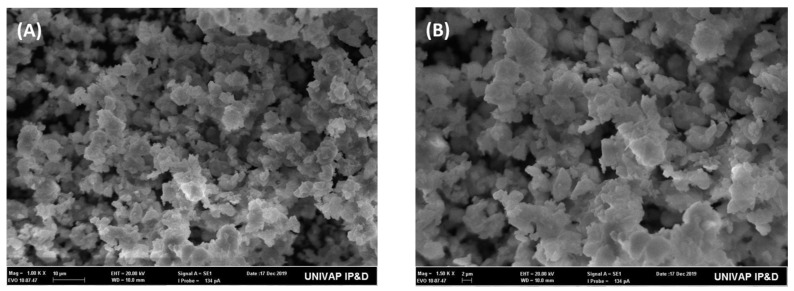
Representative SEM images of bioactive glass particles. (**A**) General view of particles at 1000×magnification (bar = 10 µm); (**B**) Agglomerates and individual particles observed at 1500× magnification (bar = 2 µm).

Figure 3A,B shows scanning electron microscopy (SEM) micrographs of bioactive glass powder, demonstrating particles with irregular morphology, angular edges, and a rough surface. Agglomerates formed by smaller particles, with varying micrometer sizes are also observed. The morphology obtained is characteristic of amorphous bioactive glasses, resulting in high surface area and potential biological reactivity.

### 3.4. Quantitative Analysis of SEM

Quantitative analysis of the SEM micrographs confirmed the presence of small agglomerates with submicron dimensions (Table 4). For the bioactive glass powder at 1000× magnification (scale bar: 10 µm), the projected equivalent circular diameter (ECD) exhibited a median of 0.108 µm (D10: 0.071 µm; D90: 0.333 µm). At higher resolution (1500× magnification, scale bar: 2 µm), smaller structures were observed, with a median ECD of 0.020 µm (D10: 0.014 µm; D90: 0.045 µm).

**Figure 4:**
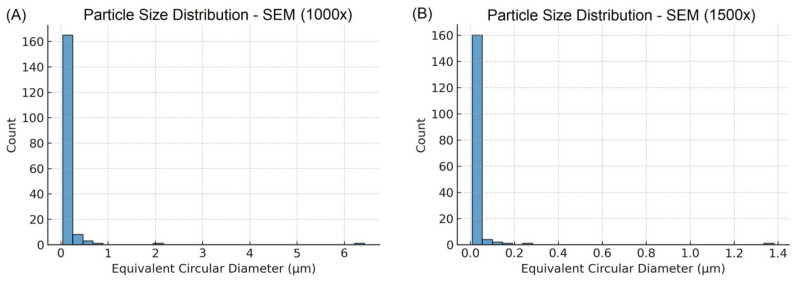
Particle size distributions. (**A**) 10 µm scale, 1000× magnification; (**B**) 2 µm scale, 1500× magnification.

The mean ± SD values were 0.233 ± 0.761 µm for the 1000× images and 0.042 ± 0.163 µm for the 1500× images. These data indicate that the material is predominantly composed of submicron agglomerates, with relatively narrow distributions, in agreement with the qualitative SEM observations. The particle size distributions are presented as histograms in Figure 4A,B.

## 4. Discussion

Orthodontic patients are at an increased risk of developing early caries during multibracket therapy, with WSL representing the earliest clinical signs of dental caries formation [7,11]. Preventing and treating enamel demineralization remain significant challenges for dentists, with early detection and remineralization being key management strategies [33]. Biomaterials capable of releasing calcium can aid in preventing and arresting the progression of initial caries lesions, such as WSL, by releasing calcium and phosphate precipitating minerals into the micropores of enamel prisms, filling gaps caused by demineralization, and restoring mineral content [7,34].

Bioactive glass materials can mechanically and chemically bond to enamel and dentin and have biomimetic properties when immersed in body fluids leading to the formation of tooth-like hydroxyapatite [35,36]. Despite these properties, the impact of these biomaterials on bond strength after their incorporation into resin-based materials is not yet completely understood. In this study, the incorporation of different bioactive glass particles concentrations in a light-cure orthodontic adhesive produced distinct shear bond strength values, so the first null hypothesis was rejected.

The light-cure orthodontic adhesive Transbond XT exhibits high shear bond strength and is widely used in orthodontic research, which justifies its selection for this study [7,37,38,39]. Hellak et al. [40] reported favorable results when evaluating the shear bond strength of two self-etching adhesives on various prosthetic surfaces and enamel, with the Transbond XT total etch system demonstrating the highest shear bond strength to human enamel. Corroborating these findings, in this study, the light-cured orthodontic adhesive was also used with the total etch approach and exhibited the highest values of shear bond strength in comparison to the other tested groups.

Differently, when the bioactive glass particles were incorporated into the light-cured orthodontic adhesive, lower values of bond strength could be observed. Although the TXT30 and TXT50 groups showed a decrease in bonding strength, the TXT20 group, with 20% wt of bioactive glass incorporated, exhibited values statistically similar to the FLB group, which is the commercial adhesive FL Bond II (SHOFU) with S-PRG (Surface Pre-Reacted Glass) particles incorporated in its formulation.

S-PRG particles are formed by a surface-pre-reacted ionomeric glass, resulting in a three-layer structure. They release multiple ions, including strontium (Sr^2+^), boron (BO_3_^3−^), fluoride (F^−^), sodium (Na^+^), silicate (SiO_3_^2−^), and aluminum (Al^3+^) in high concentrations [41]. This composition is designed to provide a variety of biological and physicochemical effects. This multiple release confers properties such as acid neutralization, bacterial inhibition, mineralization promotion, tooth strengthening, and modulation of cellular activity.

A possible explanation for the FL Bond II performance is the chemical bonding to the inorganic content of the enamel by the S-PRG particles. S-PRG particles bonds to enamel through an acid-base reaction and, the release of ions such as fluoride can buffer lactic acid and prevent enamel demineralization [41]. TXT20 group possibly presented similar shear bond strength because of bioactive glass properties of calcium and phosphate release that can reduce microleakage by promoting dental surface remineralization and the enhancement of the modulus of elasticity of the adhesive [7,34]. These characteristics make bioactive glass a favorable component in dental adhesives. These findings corroborate the results found by Zeinab et al. [42] in alignment with those observed in the TXT20 group for the shear bond strength test.

Additionally, the shear bond strength values observed in the TXT30 and TXT50 groups remain clinically acceptable, as the literature has shown that shear bond strength ranging between 5 and 8 MPa is sufficient to prevent adhesive failure of orthodontic brackets [42,43,44]. Furthermore, it is important to consider orthodontic dynamics, intraoral conditions, materials, technique and procedures when extrapolating in vitro test results to clinical reality.

The ARI data indicated that only in the TXT group was the composite entirely bonded to the tooth (score 3). In contrast, a single sample from the TXT50 and FLB groups exhibited a score of 0, indicating no remaining composite on the tooth surface. The ARI is a commonly used metric to quantify the adhesive residue left on the tooth after debonding [45]. According to the study conducted by Cervantes-Ganoza [46], an Adhesive Remnant Index (ARI) score of “0” is commonly associated with enamel damage. This association is attributed to the increased stress exerted on the enamel surface during the debonding process, which can lead to greater structural compromise. Ideally, an orthodontic biomaterial should present a mixed or cohesive failure pattern—reflected by ARI scores of “1” or “2”—as these outcomes are generally indicative of more favorable bonding characteristics and are consistent with the findings of the present study.

In this study, the degree of conversion (DC) was also evaluated. The highest DC values were observed in the TXT20 and FLB groups, leading to the rejection of the second null hypothesis. Lower DC values may negatively affect polymer stability and the adhesive’s resistance to chemical degradation, thereby influencing the mechanical performance of the composite material [47]. Additionally, insufficient monomer conversion can compromise the mechanical properties of the adhesive resin and accelerate polymer degradation [48,49]. Previous studies [49,50,51] have indicated that incorporating bioactive glass fillers into adhesive formulations can enhance the degree of conversion. Inorganic glass particles are capable of diffracting light, which increases the activation of photoinitiator molecules and promotes polymerization among monomers [47]. However, excessive amounts of bioactive glass may hinder polymerization due to the particle size, which can restrict monomer mobility. Smaller particles with greater surface area are more favorable to the polymerization process [52]. These observations are consistent with the results of the present study, in which the highest degree of conversion values were observed in the TXT20 experimental group and the FLB group.

The calcium release test was conducted following the methodology described by Hannig et al. [32,33]. Significant differences were observed in calcium concentration released by the experimental groups, and then, the third null hypothesis was rejected. The TXT50 group exhibited the highest calcium release values, which can be attributed to its high bioactive glass content. This bioactive glass consists of 45 wt% of silicon dioxide (SiO_2_), 24.5 wt% of calcium oxide (CaO), 24.5 wt% of sodium oxide (Na_2_O), and 6 wt% of phosphorus pentoxide (P_2_O_5_). The TXT20 group showed results similar to those of the FLB group, which contains SPR-G particles. These particles are composed of fluoride, strontium, borate, aluminum, and sodium, but lack calcium [53]. The use of simplified adhesive systems is justified by the development of new formulations aimed at reducing the number of clinical steps in the adhesion process, thereby minimizing treatment time [40].

Bioactive glass particles exhibit remineralizing properties by releasing calcium and phosphate ions, neutralizing acids, and increasing pH. Their ability to release ions upon dissolution in biological fluids imparts antibacterial and neutralizing properties, facilitating the formation of a mineral matrix analogous to natural hydroxyapatite [17,18,20,21,22,53]. The deposition of calcium on demineralized enamel may prevent the reduction in bond strength at the resin/enamel interface [37]. The S-PRG particles technology in the FL Bond II adhesive improves the resistance to pH variations by forming fluoride-apatite and strontium-apatite [51]. This mechanism might explain the elevated calcium release observed in the FLB group, comparable to the TXT20 group, despite the absence of calcium ions in the FL Bond II adhesive composition.

The TXT group was used according to the manufacturer’s recommendation, without bioactive glass-incorporated, so it presented lower results regarding the releasing of ions. The data demonstrated that the addition of bioactive glass particles to the adhesives increased calcium release proportionally to the incorporation concentration by weight. Calcium release helps to minimize the demineralization process of the tooth enamel structure, since calcium can neutralize the demineralizing effects of acids metabolized by bacteria and contribute to increasing the pH of the oral environment [9,54].

It has been reported that the tested bioactive glass presents acid-neutralizing properties which is an effective material for preventing WSL during orthodontic treatment [42]. Mechanical retention of biofilm, combined with poor hygiene around orthodontic brackets and a cariogenic diet, can lead to an increase in cariogenic bacteria around orthodontic brackets. Patients with high levels of *Streptococcus mutans* and *Lactobacillus* before treatment are at greater risk of developing caries lesions during orthodontic treatment [54]. These cariogenic bacteria reduce the pH through the metabolization of fermentable carbohydrates and the subsequent release of acids, which in turn causes the release of calcium and phosphate from enamel hydroxyapatite, contributing to the formation of WSL [13,14,54].

If an association can be established between the variables analyzed, neither the TXT20 nor the FBL group adversely affected the mechanical properties of the material in terms of bonding strength. Thus, the incorporation of 20% wt bioactive glass seems to be a suitable option for orthodontic adhesives, as it maintains bioactivity while minimizing any adverse effects on the material’s mechanical properties [49]. Furthermore, this study suggests that the bioactive glass-doped light-cured orthodontic adhesives could aid in preventing enamel demineralization through calcium release. However, it is important to note that the lack of artificial aging protocols, such as thermo-cycling or water storage, is a limitation of this study, as it hinders the extrapolation of data to clinical practice and other adhesive approaches should be tested in further studies, and the modified light-cured orthodontic resin can be tested under cariogenic conditions to confirm its protective effect. Also, future studies should include comprehensive ion release analyses, incorporating both calcium and phosphate measurements, to provide a more conclusive evaluation of hydroxyapatite formation and the long-term remineralization potential of the material.

## 5. Conclusions

The incorporation of distinct bioactive glass particle concentrations influenced the shear bond strength, degree of conversion, and calcium release. While the 50 wt% bioactive glass group exhibited the highest calcium release, both the 20 wt% of bioactive glass group and the positive control group exhibited the highest degree of conversion without compromising the bonding strength. The addition of 20 wt% bioactive glass offers a favorable balance between adhesive performance and bioactivity, supporting its potential as a viable strategy for clinical prevention of WSLs. The compromised degree of conversion and bond strength of the incorporation of 50 wt% bioactive glass represents a significant limitation for clinical use without further validation. Future studies should validate these findings under aging and simulated cariogenic conditions.

## Figures and Tables

**Table 1:** Composition of the tested materials.

Material	Composition	Manufacturer
Transbond XT	Bis-GMA, TEGDMA, 4(DINETHYLAMINO)BENZOETHANOL, Camphorquinone, Hydroquinone	3M ESPE, St. Paul, MN, USA
FL BOND ll	UDMA, TEGMA and S-PRG glass particles	Shofu Inc., Kyoto, Japan
Vitryxx® Bioactive Glass Powder G018144	45 wt% of SiO_2_, 24.5 wt% of CaO, 24.5 wt% of Na_2_O, and 6 wt% of P_2_O_5_	SCHOTT AG, Landshut, Bayern, Germany

Bis-GMA = bisphenol a diglycidyl dimethacrylate; UDMA = urethane dimethacrylate; TEGDMA = Triethylene glycol dimethacrylate; SiO_2_ = silicon dioxide; CaO = calcium oxide; Na_2_O = sodium oxide; P_2_O_5_ = phosphorus pentoxide.

**Table 2:** Mean and (standard deviation) values of DC (in percentage), shear bond strength (MPa), and calcium release (mg/L) values.

Groups	MPa (±SD) *	% DC (±SD) *	Calcium Release (±SD) *
TXT	19.50 ± 1.40 ^A^	60.28 ± 1.06 ^B^	0.14 ± 0.00 ^A^
TXT20	18.22 ± 1.04 ^AB^	73.02 ± 3.33 ^A^	0.55 ± 0.00 ^B^
TXT30	14.48 ± 1.46 ^C^	58.84 ± 4.06 ^B^	0.74 ± 0.00 ^C^
TXT50	14.13 ± 1.02 ^C^	40.67 ± 1.21 ^C^	2.23 ± 0.11 ^D^
FLB	17.62 ± 1.45 ^B^	68.50 ± 1.09 ^A^	0.47 ± 0.04 ^B^

* Different upper-case letters show significant differences between the groups (differences in columns).

**Table 3:** Number of teeth presenting adhesive remnant index (ARI) scores.

Groups	Scores				Total
	0	1	2	3	
TXT	0	2	7	1	10
TXT20	0	5	5	0	10
TXT30	0	6	4	0	10
TXT50	1	7	2	0	10
FLB	1	4	5	0	10
Total	2	24	23	1	50

TXT—(0% wt of bioactive glass-incorporated—negative control); TXT20—(20% wt of bioactive glass-incorporated); TXT30—(30% wt of bioactive glass-incorporated); TXT50—(50% wt of bioactive glass-incorporated); FLB—(positive control—FL BOND II adhesive system with S-PRG biomaterial, SHOFU Inc.); score 0—no remaining amount of composite adhered to the tooth; score 1—less than half the amount of composite adhered to the tooth; score 2—more than half the amount of composite adhered to the tooth; and score 3—all the composite adhered to the tooth amount of composite adhered to the tooth; (*p* = 0.0245).

**Table 4:** Summary of quantitative analysis of particle size distribution from SEM micrographs.

Image	Mean (±SD)	Median [IQR] (µm)	D10 (µm)	D50 (µm)	D90 (µm)
SEM (Figure 3A)	0.233 ± 0.761	0.108 [0.071–0.333]	0.071	0.108	0.333
SEM (Figure 3B)	0.042 ± 0.163	0.020 [0.014–0.045]	0.014	0.020	0.045

Data are expressed as mean ± standard deviation (SD), median [interquartile range, IQR], and percentiles D10, D50, and D90 of equivalent circular diameter (ECD).

## Data Availability

Data available on request.
